# Transbronchial Mediastinal Cryobiopsy Guided by Endobronchial Ultrasound in Addition to Endobronchial Ultrasound-Guided Transbronchial Needle Aspiration in the Diagnosis of Ilo-Mediastinal Lymphadenopathy Without Obvious Primary Lung Neoplastic Lesion: A Prospective Multicenter Study

**DOI:** 10.3390/jcm14207407

**Published:** 2025-10-20

**Authors:** Francesco Todisco, Filippo Patrucco, Aleksandar Veljkovic, Gian Carlo Vallese, Davide Indellicati, Letizia Valsecchi, Luca Riberi, Giuseppe Ielo, Paola Rebecca Iovine, Martina Ubaldi, Francesco Gavelli, Massimo Comune

**Affiliations:** 1 Interventional Pulmonology Unit, Specialistic Medical Department, AOU AOU San Luigi Gonzaga, 10043 Orbassano, Italym.comune@sanluigi.piemonte.it (M.C.); 2 Respiratory Diseases Unit, Medical Department, AOU Maggiore Della Carità Hospital, 28100 Novara, Italy; 3 Respiratory Diseases Unit, Medical Department, ASL CN2, 12060 Verduno, Italy; dindellicati@aslcn2.it; 4 Department of Translational Medicine, Università Degli Studi del Piemonte Orientale, 28100 Novara, Italy

**Keywords:** endobronchial ultrasound-guided transbronchial needle aspiration (EBUS-TBNA), endobronchial ultrasound-guided transbronchial mediastinal cryobiopsy (EBUS-TMC), cryobiopsy, mediastinal lymphadenopathy

## Abstract

**Background:** Endobronchial ultrasound-guided transbronchial needle aspiration (EBUS-TBNA) represents the gold standard technique for the diagnosis and staging of lung carcinoma. However, its diagnostic yield may be limited for granulomatous pathologies or rare tumors requiring comprehensive histological and molecular analysis. Endobronchial ultrasound-guided transbronchial mediastinal cryobiopsy (EBUS-TMC) is an emerging technique that allows for the acquisition of larger histological samples with preserved tissue architecture. The primary objective of this prospective multicenter study was to evaluate the potential increase in diagnostic yield afforded by the addition of EBUS-TMC to EBUS-TBNA in patients selected by pre-test clinical–radiological criteria presenting with hilar–mediastinal lymphadenopathies without an obvious primary neoplastic lung lesion. **Methods:** The study prospectively enrolled 91 patients across two Italian interventional pulmonology units. Patients were selected based on a pre-defined clinical–radiological criterion aimed at excluding primary lung cancer suspects. For each patient, both EBUS-TBNA and EBUS-TMC were performed. **Results:** The overall diagnostic yield for the combined procedure was 84.6% (77/91 patients). EBUS-TMC achieved a diagnostic yield of 78.0%, which was significantly higher than EBUS-TBNA alone of 60.4%. The addition of EBUS-TMC resulted in a statistically significant increase in diagnostic yield of 17.6%. This increase was particularly pronounced for lymphoproliferative diseases. The safety profile of EBUS-TMC was favorable without major complications. The clinical–radiological criterion used to select patients for EBUS-TMC in addition to the standard of care of EBUS-TBNA, aiming at excluding primary lung cancer, was correct in 89.0% of the study population (81 out of 91 patients). **Conclusions:** EBUS-TMC is a safe and feasible technique that significantly enhances the diagnostic yield (17.6% absolute increase in diagnostic yield) in patients with hilar–mediastinal lymphadenopathies. The clinical–radiological criterion employed proved effective in pre-selecting patients who benefit most from cryobiopsy, thereby enabling a more rational allocation of healthcare resources.

## 1. Introduction

EBUS-TBNA represents the gold standard technique in the diagnostic-staging process of lung cancer. The performance of biomolecular investigations has led to an increasing need to obtain substantial and high-quality material [1]. Although EBUS-TBNA has adequate yield for diagnosis and for performing the aforementioned investigations in primary lung neoplasms, it is a technique that allows for the collection of limited amounts of material, sometimes insufficient for the diagnosis of rare mediastinal tumors, lymphoproliferative diseases, or benign diseases that often require histological characterization. Endoscopic ultrasound transbronchial needle aspiration (EBUS-TBNA) remains one of the main techniques for sampling suspicious lymph nodes for granulomatous pathology; however, several studies have shown that EBUS-TBNA fails to find granulomas in about 20% of cases [2,3]. In order to obtain intact samples of larger size for histological evaluation in rare mediastinal tumors and benign pathologies or for biomolecular investigations in lung cancer, the eco-guided sampling of ilo-mediastinal lymph nodes and mediastinal lesions using a cryoprobe has recently been introduced. This technique has undergone a standardization process of indications/contraindications and execution techniques [4]. The contraindications are the same as for EBUS-TBNA. Due to the risk of bleeding extrapolated from the experience with parenchymal cryobiopsies for obtaining larger tissue fragments, it is emphasized the need to verify the lymph node vascular pattern sonographically [4]. Indications for EBUS-TMC are the evaluation of suspected lymphoproliferative diseases (both for de novo diagnosis and for recurrence diagnosis), suspected benign granulomatous process (sarcoidosis, silicosis, rare mediastinal infections, and lymph node tuberculosis), diagnosis of suspected metastases from other non-pulmonary or rare tumors (e.g., seminoma, thymoma, thymic carcinoma), new sampling of previous non-diagnostic EBUS-TBNA, sampling of necrotic lymph nodes/lesions, NSCLC stage III-IV for complete immunohistochemical and molecular analysis, mediastinal restaging after induction chemotherapy and/or radiotherapy for locally advanced NSCLC [4]. It is necessary to emphasize that a first proposal for standardizing indications in the literature was very recent and therefore the authors suggest adapting the use of this technique to the expertise and resources available in each interventional pulmonology unit. Regarding the use of cryobiopsies in sampling mediastinal and hilar lymphadenopathy or lesions, the current literature data confirm the quality of the samples and the absence of significant artifacts. In the context of lung cancer, where cryobiopsies can be used in specific situations where it is essential to obtain adequate material for molecular investigations, the amount of extracted DNA and RNA is much higher than that obtained from traditional EBUS-TBNA cytospins. Freezing does not pose significant problems for the pre-analytical and analytical phases of this process [5].

The purpose of the study is to evaluate the ability of a clinical–radiological criterion already in use at the Interventional Pulmonology Units of the San Luigi Gonzaga University Hospital in Orbassano and the Respiratory Diseases Unit of the Maggiore della Carità University Hospital in Novara to identify patients who benefit most from the addition of eco-guided mediastinal cryobiopsy (EBUS-TMC) to traditional eco-guided transbronchial needle aspiration (EBUS-TBNA).

The primary objective is to evaluate the potential increase in diagnostic yield due to the addition of EBUS-TMC to EBUS-TBNA in patients who underwent EBUS-TMC for ilo-mediastinal lesions without evidence of primary lung tumor on CT and/or PET-CT imaging within a year. Secondary objectives of the study are to evaluate the safety profile, duration, and technical characteristics of EBUS-TMC procedures.

## 2. Materials and Methods

### 2.1. Patients: Inclusion Criteria

All patients undergoing bronchial endoscopic ultrasound for cytological or histological sampling for the diagnosis of hilar–mediastinal lesions at the Interventional Pulmonology Unit of the San Luigi Gonzaga University Hospital in Orbassano and the Respiratory Diseases Unit of the Maggiore della Carità University Hospital in Novara were evaluated for possible indication for EBUS-TMC. The study was approved by the local ethical committee (ethics committee protocol n. 0002469, 09 January 2024) and was conducted in accordance with the Declaration of Helsinki.

The indication for the EBUS procedure was determined by the physician in charge of the patient (usually an oncologist, pulmonologist, or internist) and was related to the need for cytological–histological diagnosis of hilar–mediastinal lymphadenopathy detected on a PET or CT scan, according to guidelines or established clinical indications. This indication was reassessed and potentially confirmed by the interventional pulmonologist performing the procedure, who evaluated the actual indication, utility, and absence of contraindications. All patients had a recent chest CT or PET-CT scan (performed within 3 months before the procedure).

At the time of writing the study protocol, as there were no guidelines or clinical indications available on when to use EBUS-TMC in addition to EBUS-TBNA compared to EBUS-TBNA alone, the interventional pulmonologist selected patients eligible for EBUS-TMC (from the total number of patients undergoing EBUS diagnostics) based on the characteristics of the CT or PET-CT imaging. We included patients over 18 years old, with hilar–mediastinal lymphadenopathy without parenchymal consolidation of suspected primary lung neoplasms were selected for EBUS-TBNA and subsequent EBUS-TMC.

This group of patients was subclassified as follows:

- Group 1: absence of parenchymal lung consolidations;
- Group 2: diffuse or multifocal interstitial involvement;
- Group 3: patients with focal lung consolidations not suspicious for primary lung neoplasms.

Patients with mediastinal and hilar lymphadenopathy with parenchymal consolidation of suspected primary lung neoplasms underwent exclusive EBUS-TBNA and were not included in the study.

### 2.2. Patients: Exclusion Criteria

Patients were excluded from the study if they

- were under 18 years of age;
- had endobronchial/endotracheal tumors diagnosed by endobronchial biopsy;
- already had a neoplastic diagnosis (primary lung or other) and were proposed for EBUS-TBNA for staging purposes.

### 2.3. Study Duration

We conducted a multicenter prospective observational study; enrolment started in 10 January 2024 and lasted for 12 months.

### 2.4. Characteristics of the EBUS-TMC Procedure

The procedure for transbronchial lymph node cryobiopsy guided by endobronchial ultrasound (EBUS-TMC) as performed in the study centers is described as follow. The procedure was performed under deep sedation with anesthesiologist assistance, after the placement of a laryngeal mask or endotracheal tube, or under conscious sedation managed by the interventional pulmonologist performing the endoscopic procedure, similar to routine EBUS-TBNA procedures. EBUS may or may not be preceded by an inspecting bronchoscopy using a flexible video bronchoscope to exclude endoscopic abnormalities suggestive of pathology requiring biopsy. Subsequently, the hilar–mediastinal lymph nodes were evaluated using endoscopic ultrasound. The following endoscopes were used: Pentax model EB-1970UK at the Interventional Pulmonology Unit of the San Luigi Gonzaga University Hospital in Orbassano and Olympus model BF-UC190F at the Respiratory Diseases Unit of the Maggiore della Carità University Hospital in Novara. The operator decided which lymph node stations to sample with EBUS-TBNA and EBUS-TMC based on accessibility, size, and morphological characteristics of the lymph node. If multiple lymph node stations were suitable, at least 2 stations were usually sampled.

As indicated by Ariza-Prota et al. [4], EBUS-TBNA was performed first on the selected lymph node station.

The needles for EBUS-TBNA used are reported in the Supplementary Materials (Supplementary Table S1).

The type of needle used for each sampling was recorded.

Following the execution of EBUS-TBNA and the creation of the tunnel through the mucosa, submucosa, and lymph node capsule, in accordance with the description by Ariza-Prota [4] and detailed in the introduction, EBUS-TMC was performed. For this purpose, the cryoprobe was inserted into the lymph node station using the access route previously created by the needle. If insertion of the cryoprobe into the lymph node was not possible, to optimize the success of the cryobiopsy, an additional pass was made using the same needle or an alternative type of needle (this eventuality was documented). Electrocautery or similar devices are not used to facilitate the insertion of the cryoprobe into the lymph node.

The EBUS-TMC procedure was performed as described in the literature: real-time visualization of the cryoprobe, freezing time of 5–7 s, retraction of the echoendoscope and cryoprobe en bloc, and sample collection. Typically, at least 2 cryobiopsies were performed for each lymph node station.

The cryobiopsy system used was: ERBE-CRYO 2 (Erbe Elektromedizin GmbH, Tübingen, Germany), using dedicated flexible cryoprobes, single-use, with a diameter of 1.1 mm and a length of 1150 mm, produced by the same company.

To maximize the diagnostic success of the procedure, if deemed necessary by the interventional pulmonologist, patients may have undergone EBUS-TBNA in additional lymph node stations beyond those sampled in TMC.

### 2.5. Data Collection

For each procedure, the following information was recorded:

- Age, sex, date of the procedure.
- Type of procedure performed.
- Duration of the procedure.
- Characteristics of the CT/PET-CT imaging. Patients with hilar–mediastinal lymphadenopathy without parenchymal consolidations of suspected primary lung neoplasms were classified in three groups as specified above.Type and level of sedation.
- Presence of laryngeal mask or endotracheal tube.
- Endoscopic findings with possible identification of endobronchial lesions suggestive of neoplasia and site of biopsy.
- Significant periprocedural medical complications leading to procedure suspension (e.g., significant arrhythmias, acute pulmonary edema, severe hypoxemia, cardiac arrest); bleeding (classified as mild–moderate if it did not lead to procedure suspension, or massive if the sampling procedure was stopped, and how it was managed).
- Number and location of lymph node stations sampled with EBUS-TBNA and EBUS-TMC.
- Number and type of needles used.
- Number of cryobiopsy samples.
- Number of cryoprobes used.
- Outcome of the cryobiopsy: successful (sample obtained) or failed (no sample obtained) with documentation of any technical complications.
- Any complications: pneumothorax (usually assessed by thoracic ultrasound before patient discharge or alternatively by chest X-ray); presence of fever, hospitalization, or emergency department visit for any reason within 48 h of the procedure.

### 2.6. Cytological and Histological Analysis

The TBNA and TMC samples obtained were sent to the pathology laboratory of the San Luigi Gonzaga University Hospital and the pathology laboratory of the Maggiore della Carità University Hospital for cytological analysis (for TBNA samples) and histological analysis (for TMC samples). The pathology laboratories provide two separate and independent reports: one cytological report obtained from the sample collected by EBUS-TBNA and one histological report obtained from the sample collected by cryobiopsy (EBUS-TMC).

On both reports, the pathologist indicates, as per clinical practice, the adequacy or inadequacy of the sample for diagnostic purposes, based on the presence or absence of representative tissue from the sampling site (e.g., lymphocytes or lymphoid tissue in the case of lymph node sampling), the diagnosis, and the type of diagnosis (descriptive or definitive).

For the study, the results of the cytological and histological reports received from the pathology department were classified based on

- Adequacy or inadequacy of the sample for a diagnostic judgment;
- Definitive/non-definitive diagnosis;
- Type of definitive diagnosis obtained.

### 2.7. Statistical Analysis

The diagnostic yield (defined as the number of conclusive diagnoses obtained among the patients undergoing the procedure) of EBUS TBNA and EBUS-TMC was evaluated; the difference between the diagnostic yields of the two methods will be calculated, and the significance of this result was assessed. Based on the histopathological assessment, the type and frequency of diagnoses obtained through the various sampling methods were described. The frequency of any complications and the average duration of EBUS-TMC procedures were also listed. Statistical analyses were performed using SPSS software (IBM SPSS software statistics v28). The data are presented descriptively. All continuous variables are presented as mean ± standard deviation. Categorical variables, including diagnostic yield, are presented as frequency and percentage. Continuous data were compared between groups using Student’s *t*-test for parametric variables and the Mann–Whitney U test for non-parametric variables. Categorical data were compared using the chi-square test or Fisher’s exact test depending on the observed frequencies. All analyses were conducted with two-tailed tests, and a *p*-value less than 0.05 were considered significant.

According to previously published definitions [6], diagnostic yield is defined as the proportion of all individuals undergoing the diagnostic procedure under evaluation in whom a specific malignant or benign diagnosis is established. The numerator includes all patients with suspected lymphadenopathies in whom the result of a minimally invasive diagnostic procedure establishes a specific benign or malignant diagnosis that is sufficient to inform patient care, and the denominator should include all patients in whom the minimally invasive procedure was attempted or performed. Diagnostic yield increase is defined by comparing diagnostic yield obtained by different sampling techniques. Overall diagnostic yield is defined by considering the total amount of patients undergoing the sampling procedure; disease-stratified diagnostic yield is defined by considering the diagnosis obtained at end of clinical and radiological follow-up period: this definition accounts for diagnosis obtained by multidisciplinary team discussion and additional biopsies.

#### 2.7.1. Sample Size Estimation

Approximately 400 patients each year are referred to the Interventional Pulmonology Unit of the San Luigi Gonzaga University Hospital in Orbassano (TO) and the Respiratory Diseases Unit of the Maggiore della Carità University Hospital in Novara for transbronchial hilar–mediastinal biopsies guided by endoscopy (EBUS). The majority of these patients (about 85%) have a clinical–radiological suspicion of lung cancer and undergo the traditional EBUS-TBNA procedure exclusively. The remaining 15% of patients do not have radiological evidence of primary lung neoplasms and are therefore subjected to EBUS-TMC following EBUS-TBNA. From the available literature [2,3,7], it is evident that in the population of patients with hilar–mediastinal lymphadenopathy not secondary to lung cancer, the diagnostic yield of EBUS-TBNA is 53.2%, while the diagnostic yield of EBUS-TMC is 80.9%.

For statistical relevance, based on the assumption that the diagnostic yield of EBUS-TMC in patients with mediastinal lymphadenopathy and non-suggestive imaging for primary lung cancer is comparable to patients with cytological–histological diagnoses other than lymph node metastasis of lung cancer (80.9%), the sample size was calculated considering a type I error of 0.05 and a type II error of 20%, equivalent to 80% power for two independent groups, resulting in 44 patients per EBUS-TMC and EBUS-TBNA group. Since each patient undergoing EBUS-TMC also undergoes EBUS-TBNA, each patient can be included in both groups, leading to an estimated total sample size of 44 patients per center; this estimate is valid for both centers; thus, the expected total sample size is 88 patients. This sample size is easily achievable within the one-year study duration.

## 3. Results

### 3.1. Study Population

The study included 91 EBUS-TMC procedures: 59 at the Interventional Pulmonology Unit of the San Luigi University Hospital in Orbassano (64.8%) and 32 at the Respiratory Diseases Unit of the Maggiore University Hospital in Novara (35.2%). The radiological presentation on chest CT or whole-body PET-CT that led to the indication for EBUS-TMC was Group 1 in 39 patients (42.8%), Group 2 in 27 patients (29.7%), and Group 3 in 25 patients (27.5%). Demographic data are presented in Table 1 (Table 1).

### 3.2. Procedure

The average duration of the procedure was 51 ± 15 min. The procedure was performed in 29 cases (31.9%) under conscious sedation managed by the interventional pulmonologist, and in 62 cases (68.1%) under deep sedation managed by a dedicated anesthesiologist. In most of the cases with deep sedation, a laryngeal mask was chosen for airway access and ventilation (54 cases), while in a smaller number of cases, an endotracheal tube was used (8 cases).

The endoscopic appearance was normal in 85 patients (93.4%). In 6 patients (6.6%), the endoscopic exploration revealed a pathologically irregular mucosa (infiltrated, swollen) on which biopsies were not performed because the operator deemed them unrepresentative of the target.

Out of a total of 91 procedures, sampling attempts were made with EBUS-TMC in 146 lymph node sites (Table 2). In order of sampling frequency, the sites were 7 (69 samplings—47.2%), 11L (23 samplings—15.8%), 4R (22 samplings—15.1%), 11R (21 samplings—14.4%), 4L (5 samplings—3.4%), 10R (4 samplings—2.7%), 3P and lesion with transbronchial access into the intermediate bronchial wall (1 sampling each—0.7%). Out of these samplings, 14 were unsuccessful due to the inability to insert the cryoprobe into the lymph node after the first phase of EBUS-TBNA. The stations where this event occurred were in order 4R (5 unsuccessful attempts), 7 (4 times), 11R (2 times), 10R, 4L, and 11L (1 attempts each). In these stations, the only available histopathological data is the cytological examination on EBUS-TBNA. The unsuccessful outcome of the cryobiopsy was taken into account for statistical purposes.

In 48 procedures, sampling attempts were made in a single site (52.7%). In 32 procedures, samplings were made in two sites (35.2%). In 11 procedures, sampling attempts were made in three sites (12.1%).

In most lymph node stations, 2 cryobiopsies were performed, with an average of 2.11 ± 0.79 cryobiopsies per site. It is noted that the average number of cryobiopsies per site decreases progressively as the procedure progresses: 2.25 ± 0.75 for the first sampled site, 1.90 ± 0.82 for the second sampled site, 1.62 ± 0.51 for the third sampled site.

In 42 procedures, cytological results of EBUS-TBNA were available without a corresponding histological result of EBUS-TMC, for a total of 53 sites. They include both sites where sampling with EBUS-TMC was unsuccessful and sites sampled exclusively with EBUS-TBNA. Among these procedures, there were 4 procedures in which sampling with EBUS-TMC failed in all tested sites; therefore, TBNA was the only successfully performed biopsy. The unsuccessful outcome of the cryobiopsy in these procedures was taken into account for statistical purposes. In other procedures, additional sites were sampled with EBUS-TBNA in addition to those sampled with EBUS-TMC in order to maximize the diagnostic success of the procedure, as outlined in the study methods.

The most commonly used needle types were the 22 G caliber needle with a crowned tip in 94 sites and the 19 G caliber needle in 47 sites. It is noted that the 22 G crowned caliber needle was almost exclusively used by the Orbassano site, while the 19 G caliber needle was exclusively used by the Novara site. In three procedures, it was necessary to change the needle (choosing the second needle from the two mentioned above) in order to successfully insert the cryoprobe into the lymph node, as the first needle used did not guarantee the creation of an adequate tunnel. Additionally, in the 14 sites where the EBUS-TMC attempt was ineffective, the initial needle choice still fell on one of the two needles successfully used in other procedures (Table 2).

### 3.3. Complications

The only recorded complication was mild to moderate bleeding in 7 patients (7.7% of procedures) all managed with suction and instillation of cold saline. The extent of this event did not lead to the suspension of sampling. There were no cases of iatrogenic pneumothorax. No infectious complications were reported within 48 h of the procedure.

Other technical complications related to the procedure were recorded, in addition to the most important one of the impossibility of inserting the cryoprobe into the target. Cryoprobe breakage occurred in 4 procedures, in all cases after at least two biopsies: in 2 cases, a second cryoprobe was used to proceed with sampling from other lymph node stations, while in the other two cases, the biopsies already performed were considered sufficient. In two procedures, the sample was lost during the extraction of the echoendoscope and the en bloc probe after freezing.

### 3.4. Diagnostic Results

In the 146 sites sampled with both techniques, suitable samples were obtained in 121 sites (82.9%) with EBUS-TBNA and 118 sites (80.8%) with EBUS-TMC. Concordance between EBUS-TBNA and EBUS-TMC was found in 98 sites (67.1%). The diagnostic yield for a single site sampled with both techniques was 53.4% with EBUS-TBNA and 70.5% with EBUS-TMC. The increase in diagnostic yield was 17.1% with a gain in 26.0% of the sites (*p* = 0.002).

The definitive diagnosis was obtained with EBUS-TMC and/or EBUS-TBNA in 77 patients (overall diagnostic yield of 84.6%). In 14 patients (16%), the procedure was inconclusive in the diagnostic process: in 4 patients, a diagnosis of sarcoidosis was made (in 2 cases with subsequent mediastinoscopy, in 1 case with concomitant transbronchial lung parenchyma biopsy, in 1 case within a multidisciplinary group after clinical–radiological follow-up); in 4 patients, a diagnosis of lymphoproliferative disease was made (in 2 cases with subsequent bone marrow biopsy and in 2 cases with subsequent excisional lymph node biopsy); in 6 patients, a definitive diagnosis has not been achieved at the end of follow-up (Table 3).

The overall diagnostic yield of the EBUS-TBNA and EBUS-TMC procedures was 84.6% (77 out of 91 patients). The overall diagnostic yield of samples obtained with EBUS-TBNA was 60.4% (55 out of 91 patients) and 78.0% for EBUS-TMC (71 out of 91 patients). 22 out of 91 patients (24.1%) received a diagnosis with EBUS-TMC where EBUS-TBNA was not diagnostic. The difference between the overall diagnostic yield of the procedure and the diagnostic yield of EBUS-TMC could be identified in the 6 procedures where the sampling with EBUS-TMC was not suitable while EBUS-TBNA was suitable and diagnostic. The addition of EBUS-TMC led to an increase in diagnostic yield of 17.6% (16 patients out of 91) which reached statistical significance (*p* = 0.016) (Table 3).

The 91 patients included in the study received the following diagnoses at the end of the diagnostic process and radiological follow-up: 41 sarcoidosis (45%); 15 pneumoconiosis (16.5%) including 12 anthracosis (13.1%) and 3 silicosis (3.3%); 10 primary lung neoplasms (11%) including 5 non-small-cell lung cancers of adenocarcinoma type (5.5%), 2 non-small-cell lung cancers NOS (2.2%), 2 small-cell lung cancers (2.2%), 1 non-small-cell lung cancer of squamous cell type (1.1%); 1 typical carcinoid (1.1%); 1 lymph node metastasis from colorectal adenocarcinoma (1.1%), 1 amarthocondroma (1.1%); 13 lymphoproliferative diseases (14.3%); 2 tuberculosis (2.2%); 1 reactive lymph node in NSIP (1.1%); and 6 patients without a definitive diagnosis (6.6%) (Table 4).

The clinical–radiological criterion used to select patients for EBUS-TMC in addition to the standard of care of EBUS-TBNA, which aimed to exclude patients with primary lung neoplasms before the procedure, was correct in 89.0% of the study population (81 patients out of 91 with a diagnosis other than primary lung neoplasms).

After division into radiological groups, the diagnostic yields are as follows:

- In Group 1 (lymphadenopathies without parenchymal lung consolidations), the overall diagnostic yield of the EBUS-TBNA and EBUS-TMC procedures was 92.3% (36/39 patients): EBUS-TBNA 64.1% (25/39 patients), EBUS-TMC 84.6% (33/39 patients). It is worth noting that 4 out of 39 patients (10.2%) in this group were diagnosed with primary lung cancer.
- In Group 2 (lymphadenopathies with diffuse or multifocal interstitial involvement), the overall diagnostic yield of the EBUS-TBNA and EBUS-TMC procedures was 70.4% (19/27 patients): EBUS-TBNA 44.4% (12/27 patients), EBUS-TMC 66.7% (18/27 patients). It is worth noting that 2 out of 27 patients (7.4%) in this group were diagnosed with primary lung cancer.
- In Group 3 (lymphadenopathies with focal lung consolidations not suspicious for primary lung neoplastic lesions), the overall diagnostic yield of the EBUS-TBNA and EBUS-TMC procedures was 88.8% (22/25 patients): EBUS-TBNA 72.0% (18/25 patients), EBUS-TMC 80.0% (20/25 patients). It is worth noting that 4 out of 25 patients (16%) in this group were diagnosed with primary lung cancer.

Based on radiological appearance, benign pathologies were mostly represented in Group 1 (41.1%, in 35.7% and 23.2% of groups 2 and 3, respectively), non-lung primary neoplastic pathologies in Group 3 (50.0%, then 43.7% in Group 1 and 6.3% in Group 2) and lung tumors in half of Group 1 (50.0%, in 30.0% and 20.0%, respectively, in Group 3 and 2).

The overall diagnostic yield of the EBUS-TBNA and EBUS-TMC procedures was 86.4% with a 22 G crowned tip needle and 83.3% with a 19 G needle, with no statistically significant difference (*p* = 0.755).

The overall diagnostic yield of the EBUS-TBNA and EBUS-TMC procedures was 88.7% with the procedure conducted under general anesthesia and 75.8% with the procedure conducted under conscious sedation managed by the interventional pulmonologist, with no statistically significant difference (*p* = 0.121).

In sarcoidosis, the combined diagnostic yield of EBUS-TBNA and EBUS-TMC was 90.2%. EBUS-TBNA alone had a yield of 68.3%, while EBUS-TMC alone had a yield of 80.5%. The addition of EBUS-TMC increased the overall diagnostic yield by 12.2% but not statistically significant (*p* = 0.206). For pneumoconiosis, the combined diagnostic yield of EBUS-TBNA and EBUS-TMC was 100%. EBUS-TBNA had a yield of 73.3%, and EBUS-TMC had a yield of 93.3%. The addition of EBUS-TMC increased the diagnostic yield by 20%, although not statistically significantly (*p* = 0.329).

In lymphoproliferative pathologies, the combined diagnostic yield of EBUS-TBNA and EBUS-TMC was 69.2%. EBUS-TBNA had a yield of 23.1%, while EBUS-TMC had a yield of 69.2%. The addition of EBUS-TMC increased the diagnostic yield by 46.1% (*p* = 0.047).

For non-hematologic neoplastic pathologies, the combined diagnostic yield of EBUS-TBNA and EBUS-TMC was 100%. EBUS-TBNA had a yield of 33.0%, and EBUS-TMC had a yield of 100%. The addition of EBUS-TMC increased the diagnostic yield by 23.0% (*p* = 0.223). In primary lung tumors, the combined diagnostic yield of EBUS-TBNA and EBUS-TMC was 100%. EBUS-TBNA had a yield of 90.0%, while EBUS-TMC had a yield of 100%. The addition of EBUS-TMC increased the diagnostic yield by 10.0% (*p* = 1.000).

For metastatic or rare neoplasms, the combined diagnostic yield of EBUS-TBNA and EBUS-TMC was 100%. EBUS-TBNA had a yield of 33.3%, while EBUS-TMC had a yield of 100%. The addition of EBUS-TMC increased the diagnostic yield by 66.7% (*p* = 0.400).

In tuberculosis, the combined diagnostic yield of EBUS-TBNA and EBUS-TMC was 100%. Both procedures were diagnostic in a patient with reactive lymph nodes.

## 4. Discussion

Our study confirms the feasibility of lymph node sampling using EBUS-TMC, ensuring an increase in diagnostic yield compared to conventional EBUS-TBNA. The sampling procedure with EBUS-TMC has a safety profile entirely overlapping with that of EBUS-TBNA. The procedure can be performed either under general anesthesia or under conscious sedation. A slight diagnostic yield increase is found in the procedures performed under general anesthesia compared to those performed under conscious sedation but this difference does not reach statistical significance. The most commonly used needle types in our case series (22 G crowned tip needle and 19 G needle) are both valid for sampling and creating the tunnel for cryobiopsy.

The decision to perform EBUS-TMCs is driven by the need for superior anatomopathological samples compared to EBUS-TBNA. EBUS-TMC offers advantages such as larger samples with preserved tissue architecture, better extraction of genetic material for molecular characterization, and immunophenotyping. While EBUS-TBNA is effective for diagnosing primary thoracic malignancies and metastatic lesions, its efficacy may vary for benign pulmonary interstitial diseases and hematological disorders. Limited sample quantity can hinder definitive diagnoses, especially for conditions like lymphoma or sarcoidosis. Studies show varying diagnostic accuracy for sarcoidosis with EBUS-TBNA [8,9,10]. Our study’s diagnostic yield of EBUS-TBNA was 60.4%, lower than reported in the literature, likely due to a high representation of benign pathologies and rare neoplasms [9,11]. Comparisons with standard approaches show similar diagnostic yields for benign hilar–mediastinal lymphadenopathy. The rigidity of sarcoid lymph nodes and architectural changes induced by the disease may contribute to diagnostic variability with EBUS-TBNA.

From a resource allocation perspective, the clinical–radiological criterion used can be considered a simple and cost-effective tool to select patients for EBUS-TMC, as the literature and our case series confirm that patients with primary lung neoplasms are those who derive the least benefit from the addition of cryobiopsy. In our selected population, EBUS-TMC provided a diagnostic yield of 78.0%, which is 17.6% higher than that of EBUS-TBNA (*p* = 0.016). Our study is the first to use a clinical–radiological criterion to pre-select patients before the procedure. Previous studies show an overall diagnostic yield of EBUS-TMC of 91.8%, 91.2%, 94%, 96% [3,7,11,12,13] which appears higher than our 78.0%. However, all previous studies include a considerable percentage of patients diagnosed with lung neoplasms (ranging from 18 to 66% of the total) compared to our incidental 11%: the presence of these patients, in whom the diagnostic yield is close to 100% (100% for EBUS-TMC), in the reported case series, makes the overall diagnostic yield higher than that reported in our study. If compared with previous studies for disease groups, diagnostic yield values vary between 80.9 and 100% for benign pathologies like our diagnostic yield of 83.9% and between 76.0 and 100% for rare tumors including lymphomas, where our diagnostic yield of 75% is slightly lower. This study provides a “real-world” yield figure for the specific patient population that stands to benefit most from the technique because, despite the limitations due to the lack of comparison with the general population undergoing EBUS-TBNA and excluded from the EBUS-TMC procedure, the most important novelty of this study is being able to pre-select patients before the procedure in whom EBUS-TMC provided a significant diagnostic advantage over EBUS-TBNA: the 17.1% increase in diagnostic yield is higher than the overall increase reported in other studies (ranging from 11.9 to 14%) [2,12]. Furthermore, considering the number of patients in whom EBUS-TMC allowed a diagnosis where EBUS-TBNA failed, this percentage rises to 24.1% (22 patients out of 91). It is important to note that, since cryobiopsy requires the preliminary creation of a tunnel with a needle, EBUS-TMC is always an additional sampling technique compared to EBUS-TBNA. The overall diagnostic yield, therefore, cannot be affected by the execution of cryobiopsies but can only be increased.

The clinical–radiological criterion used to select patients for EBUS-TMC in addition to the standard of care of EBUS-TBNA aimed to exclude patients with primary lung cancer, a group in which EBUS-TMC does not lead to a significant increase in diagnostic yield according to current literature data. Patient selection based on this simple clinical–radiological criterion aimed to pre-select patients with benign (e.g., granulomatosis and pneumoconiosis) or non-lung cancer malignancies (e.g., lymphomas, rare tumors) in whom an increase in diagnostic yield from the endoscopic procedure with EBUS-TMC is expected. This criterion was correct in 89.0% of the study population (81 out of 91 patients), but incorrect in 10 patients (11.0%), where the cytological–histological diagnosis unexpectedly turned out to be lung cancer. From these evaluations, the need to maintain a high level of suspicion for lung neoplastic pathology can be reiterated in the presence of any type of lung consolidation. However, this is not sufficient, as half of the patients with lung neoplastic diagnosis belong to the group without parenchymal lesions on CT and therefore present the least suspicious radiological picture for primary lung cancer: it can be hypothesized that to improve our selection criteria, additional tools that take into greater consideration the patient’s risk factors and medical history, in addition to predominant imaging evaluation, could be implemented.

The preliminary selection of patients, in addition to being effective from a diagnostic point of view, minimizes the procedures where offering EBUS-TMC is not only unnecessary in terms of diagnostic yield but also counterproductive in terms of economic (contingent costs related to consumables) and logistical (use of medical, nursing, and technical human resources in pulmonology and pathology units, increased procedure duration) aspects. Further studies are desirable to assess this gain.

The group of patients with neoplastic pathologies other than primary lung tumors (small-cell and non-small-cell) is the one that benefits most from the addition of EBUS-TMC with a 50% difference in diagnostic yield; our data confirm previously published one. In particular, the most significant group is that of patients with lymphoproliferative disorders: in suspicion of such pathology, the endoscopic approach has always been considered secondary but the advent of EBUS-TMC could represent a valid alternative to excisional lymph node biopsies, provided further studies consolidate the data still emerging in the literature [14,15].

In the group of patients with benign pathologies (sarcoidosis and pneumoconiosis), the overall diagnostic yield of the EBUS-TBNA and EBUS-TMC procedure was 92.8%, higher than the existing literature data on EBUS-TBNA alone [7,15,16]. Although EBUS-TMC leads to a positive trend in increase in diagnostic yield of 14.3%, this result does not reach statistical significance: this may be conditioned by the presence in this group of 5 procedures where cryobiopsy was inferior to needle aspiration. This occurrence is due to procedural or technical circumstances related to the selected target (lymph nodes with hard consistency and greater capsule–mucosa distance) that did not allow sampling of representative tissue of the lesion with the cryoprobe as obtained by EBUS-TBNA. Furthermore, the absence of a statistically significant result is probably due to the sample size, although these results suggest a promising trend.

We confirmed previously reported data that all lymph node stations were easily sampled without significant complications. A total of 4 cases out of 42 had failed sampling with EBUS-TMC due to cryoprobe insertion issues or breakage. Both crowned and 19 G needles were effective for cryoprobe insertion. In cases where EBUS-TMC attempts were unsuccessful, the choice of needle was still effective in other procedures. The lack of sampling effectiveness was attributed to procedural or technical circumstances, particularly in lymph nodes with hard consistency and greater capsule–mucosa distance, later diagnosed as sarcoidosis. Finally, the procedure’s advantage is the absence of significant complications related to either tunneling or subsequent sampling. Our study therefore confirms what has been previously reported in the literature, demonstrating that EBUS-TMC is a safe method [17,18,19].

Our study has the following limitations: data of patients excluded from EBUS-TMC based on our clinical–radiological criteria and offered only EBUS-TBNA are currently not available, This would be identified as a control group, with similar radiological findings. These data would be useful for a comparison of the diagnostic yield of the procedures in the two groups and for a comparison of the entire population eligible for mediastinal cryobiopsy, for the calculation of sensitivity, specificity, accuracy, predictive value of the criterion used for the selection of EBUS-TMC procedures. Furthermore, the presence of a control group would have given us the most faithful data on the incremental value linked to sampling using EBUS-TMC over EBUS-TBNA alone. The analysis of these data in future studies is desirable. The clinical–radiological criterion to exclude lung cancer suspects is based solely on the physician’s judgment, based on the collected medical history and available imaging. Although a selection criterion was used to select patients who underwent EBUS TMC that was effective in 89% of cases, this does not represent 100% and therefore only the definitive diagnosis allows verification of correct compliance with the inclusion criteria. For future applications, tools that use artificial intelligence with machine learning capable of making this selection should also be considered. Although the pathologists who diagnose the cytological samples from TBNA are different from those who diagnose the histological samples from TMC, another limitation of our study is the absence of a constant blinding method for the anatomopathological evaluation of the samples. It is not possible to judge whether the cytological result of TBNA or the histological result of TMC may have influenced the judgment of the pathologist analyzing the remaining samples. This may have had an impact on the study results. The inability to achieve a sufficient follow-up period for patients whose procedures were inconclusive was another limitation of our study. Lastly, given the recent introduction of the technique in the participating centers, a further future perspective could be the evaluation of the learning curve of EBUS-TMC execution and the comparison of diagnostic yields of the first procedures versus the last ones.

## 5. Conclusions

EBUS-TMC is a safe and easily practicable technique for sampling mediastinal–hilar lesions in expert interventional pulmonology centers with expertise in endobronchial ultrasound. The use of EBUS-TMC in addition to EBUS-TBNA ensures an increase in diagnostic yield in lymphoproliferative diseases, non-primary lung tumors with hilar–mediastinal involvement, and benign conditions such as sarcoidosis and pneumoconiosis. The clinical–radiological criteria used to select patients for EBUS-TMC in addition to the standard of care EBUS-TBNA has been effective in identifying patients who would benefit from cryobiopsy before the procedure. The increase in diagnostic yield in our case series is higher compared to what has been reported in the literature so far, where the presence of lung cancer patients reduces the percentage of patients who truly benefit from cryobiopsy compared to EBUS-TBNA. In order to optimize the expected diagnostic gain from cryobiopsy and allocate economic and logistic resources in a more coherent manner, it can be considered a simple and easily implementable tool in clinical practice to select patients for EBUS-TMC.

## Figures and Tables

**Table 1:** Demographic and procedures’ data.

Patients n (%)	91 (100.0%)	
Age (in years, ±SD)	57 ± 15	
Procedure duration (in min, ±SD)	51 ±15	
Sex n (%)	Male	58 (63.7%)
	Female	33 (36.3%)
Radiological pattern	Group 1	39 (42.8%)
	Group 2	27 (29.7%)
	Group 3	25 (27.5%)
Type of Sedation/Airway Management	Conscious sedation	29 (31.9%)
	Deep sedation	62 (68.1%)
	Laryngeal mask	54 (59.3%)
	Endotracheal tube	8 (8.8%)
Endoscopic appearance	Regular	85 (93.4%)
	Irregularities	6 (6.6%)
Complications	Mild to moderate bleeding	7 (7.7%)
	Severe bleeding	0 (0%)
	Pneumothorax	0 (0%)
	Infectious complications within 48 h	0 (0%)

**Table 2:** Needle type and lymph node stations sampled.

Used Needle Type	Caliber 22 G Crowned	94 (64.4%)
	Caliber 19 G	47 (32.2%)
	Caliber 22 G	5 (3.4%)
Sampled lymph node stations	7	69 (47.2%)
	11L	23 (15.8%)
	4R	22 (15.1%)
	11R	21 (14.4%)
	4L	5 (3.4%)
	10R	4 (2.7%)
	3P	1 (0.7%)

**Table 3:** Diagnostic yield divided by technique and procedural overall.

	EBUS-TBNA Diagnostic Yield	EBUS-TMC Diagnostic Yield	EBUS-TMC Diagnostic Yield Increase	Procedural Overall Diagnostic Yield	Definitive Diagnosis at the End of Follow-Up
Entire population					
Diagnostic	55/91 (60.4%)	71/91 (78.0%)	16/91 (17.6%)	77/91 (84.6%)	85/91 (93.4%)
Non-conclusive	36/91 (39.6%)	20/91 (22.0%)	*p* = 0.016	14/91 (15.4%)	6/91 (6.6%)

**Table 4:** Groups of diagnosis.

Definitive Diagnosis at the End of Follow-Up	Diagnosis Made with EBUS-TBNA	Diagnosis Made with EBUS-TMC	Diagnostic Yield Increase by Group: *p*-Value
Primary lung tumors 10/91 (11.0%)	9 (9.9%)	10 (11.0%)	1
Adenocarcinoma 5/91 (5.5%)	5 (5.5%)	5 (5.5%)	
Squamous 1/91 (1.1%)	0 (0%)	1 (1.1%)	
NOS 2/91 (2.2%)	1 (1.1%)	1 (1.1%)	
SCLC 2/91 (2.2%)	1 (1.1%)	1 (1.1%)	
Other tumors 16/91 (17.6%)	4 (4.4%)	12 (13.2%)	0.012
Lymphoma 13/91 (14.3%)	3 (3.3%)	9 (9.9%)	0.047
Carcinoids 1/91 (1.1%)	0 (0%)	1 (1.1%)	
Amarthocondroma 1/91 (1.1%)	1 (1.1%)	1 (1.1%)	
Metastasis from colorectal cancer 1/91 (1.1%)	0 (0%)	1 (1.1%)	
Benign 56/91 (61.5%)	39 (42.8%)	47 (51.6%)	0.073
Sarcoidosis 41/91 (45.0%)	28 (30.7%)	33 (36.2%)	0.206
Pneumoconiosis 15/91 (16.5%)	11 (12.1%)	14 (15.4%)	0.329
Other benign conditions 3/91 (3.3%)			
Tuberculosis 2/91 (2.2%)	2 (2.2%)	1 (1.1%)	
Reactive lymph node 1/91 (1.1%)	1 (1.1%)	1 (1.1%)	
No definitive diagnosis 6/91 (6.6%)			

NOS: not otherwise specified, SCLC: small-cell lung cancer.

## Data Availability

Data available on request due to privacy restrictions.

## Supplementary Materials

The following supporting information can be downloaded at: https://www.mdpi.com/article/10.3390/jcm14207407/s1, Table S1: EBUS TBNA needles used.
