# Supplementary material for: Transbronchial Mediastinal Cryobiopsy Guided by Endobronchial Ultrasound in Addition to Endobronchial Ultrasound-Guided Transbronchial Needle Aspiration in the Diagnosis of Ilo-Mediastinal Lymphadenopathy Without Obvious Primary Lung Neoplastic Lesion: A Prospective Multicenter Study
Source: J Clin Med. 2025 Oct 20;14(20):7407. doi: 10.3390/jcm14207407 (PMC12565598; doi:10.3390/jcm14207407)
Supplement: Supplementary file 1 [file jcm-14-07407-s001.zip › jcm-3866723-supplementary.pdf]

Supplementary Table S1. EBUS TBNA needles used:

Interventional Pulmonology Unit of the San Luigi Gonzaga University Hospital in Orbassano			
Name	Producer	Size	Type
Echotip ECHO-HD-22-EBUS-P	COOK MEDICAL LLC, Bloomington, United States	22G	Standard biopsy needle
Echotip Procore ECHO-HD-22-EBUS PC	COOK MEDICAL LLC, Bloomington, United States	22G	Standard biopsy needle
SonoTip TopGain	Medi-Globe GmbH, Rohrdorf, Germany	22G	Crown-cut tip
Respiratory Diseases Unit of the Maggiore della Carità University Hospital in Novara			
Name	Producer	Size	Type
Acquire Pulmonary Fine Needle Device	Boston Scientific Corporation, Marlborough, Massachusetts, United States	22G	Crown-cut tip
ViziShot 2 NA-U403SX-4019	Olympus, Shinjuku, Tokyo, Japan	19G	Standard biopsy needle
ViziShot NA-201SX-4021	Olympus, Shinjuku, Tokyo, Japan	21G	Standard biopsy needle
ViziShot NA-201SX-4022	Olympus, Shinjuku, Tokyo, Japan	22G	Standard biopsy needle
